# Supplementary material for: Computer-based inhibitory control training in children with Attention-Deficit/Hyperactivity Disorder (ADHD): Evidence for behavioral and neural impact
Source: PLoS One. 2020 Nov 30;15(11):e0241352. doi: 10.1371/journal.pone.0241352 (PMC7703966; doi:10.1371/journal.pone.0241352)
Supplement: S1 Flow diagram — (DOC) [file pone.0241352.s004.doc]

**
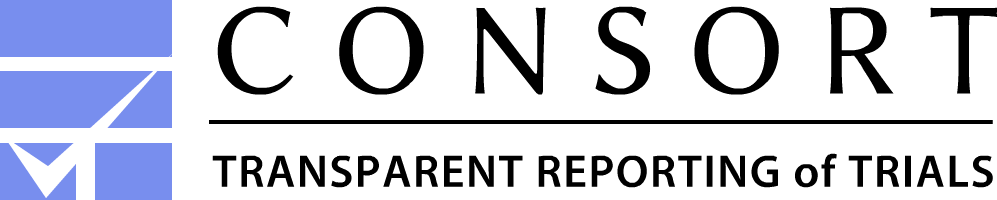
**

**CONSORT 2010 Flow Diagram**

**Allocation**

**Analysis**

**Follow-Up**

**Enrollment**

Assessed for eligibility (n= 128) )

Excluded (n= 88)

♦  Not meeting inclusion criteria (n= 30)

♦  Declined to participate (n= 50)

♦  Other reasons (n= 8)

Analysed (n= 20)
♦ Excluded from analysis (n= 0)

Lost to follow-up (give reasons) (n= 0)

Discontinued intervention (give reasons) (n= 0)

Allocated to intervention (n= 20 )

♦ Received allocated intervention (n= 20 )

♦ Did not receive allocated intervention (n= 0)

Lost to follow-up (give reasons) (n= 0)
Discontinued intervention (did not like games) (n= 1)

Allocated to intervention (n= 21)

♦ Received allocated intervention (n= 21)

♦ Did not receive allocated intervention (n= 0)

Analysed (n= 20)
♦ Excluded from analysis (n= 0)

Randomized (n= 41)
