# Supplementary material for: Computer-based inhibitory control training in children with Attention-Deficit/Hyperactivity Disorder (ADHD): Evidence for behavioral and neural impact
Source: PLoS One. 2020 Nov 30;15(11):e0241352. doi: 10.1371/journal.pone.0241352 (PMC7703966; doi:10.1371/journal.pone.0241352)
Supplement: S1 Protocol — (DOC) [file pone.0241352.s005.doc]

Part B: Experimental Design and Protocol – ALL APPLICANTS MUST COMPLETE THIS FORM

All investigators must submit a completed Part B with their New Protocol or Continuing Review application. If a protocol from a corporate sponsor or cooperative group is available, this must also be submitted.

Each question in Part B should be answered thoroughly with answers that are specific to how the research will be conducted at Children’s Hospital, Boston.

Do not cut and paste from the protocol or from a grant application to complete Part B. Instead, complete each question in Part B by referencing the applicable page and section number of the protocol which answers the questions in Part B. For some questions in Part B, such as those regarding recruitment methods, confidentiality provisions, and adverse event reporting, you will need to provide complete answers rather than references to the protocol, since the protocol will not address these items as they apply specifically to how the research will be conducted at BCH.

Further information may be obtained by referring to [the polices and procedures on the CCI website](http://www.childrenshospital.org/cfapps/research/data_admin/Site2206/mainpageS2206P3.html)

**Please provide a brief summary or abstract of this research protocol.**

*Neuroplasticity Technology for ADHD*

Attention-deficit/hyperactivity disorder (ADHD) is a paramount public health concern – impacting 3-5% of the population (Kessler et al., 2006). Although pharmacological interventions (e.g. psychostimulants) remain the main treatment for ADHD, there is an increasing need for alternative intervention strategies that offer additional angles to improve the behavioral deficits at the core of ADHD symptoms. Currently, several computer based training programs focused on training working memory have been developed to investigate this as a potential treatment, these programs have met with moderate success. However, the hypothesized central deficit in ADHD is inhibitory control, not working memory. In this study we propose a small pilot study to investigate the potential that an inhibitory control intervention will alleviate symptoms of ADHD, executive function performance in every day life, and performance on tests of executive function in the lab. The experiments in this research program investigate the feasibility of one emerging possibility: neuroplasticity-based video games that target the dysfunctional networks in the ADHD brain and harness the brain’s inherent plasticity (i.e. malleability) to strengthen the functions at the root of ADHD symptoms.

The experiments outlined in this proposal will include behavioral testing of human subjects and will be overseen by Dr. Margaret Sheridan. In both experiments, behavioral sessions will be conducted in lab facilities rented from the Laboratories of Cognitive Neuroscience (LCN) at Boston Children’s Hospital (BCH). In Expt 1, 20 participants will be asked to give consent, sign a number of forms and participate in a short 1 hour behavioral experiment and EEG recording session. In Expt 2, 50 participants will be asked to give consent, sign a number of forms and participate in a 2 hour EEG recording as they perform behavioral tasks. This session should take no longer than 2hours, with 30 minutes of setup (both consenting as well as EEG setup) and 60 minutes of behavioral assessment. Following this session in Expt 2, participants will perform the short training behavioral tasks in the comfort of their own home for 4 weeks on touch tablet computers provided at their initial baseline visit. Following the training, participants will return to BCH for a second 1.5 hour behavioral and EEG outcome assessment and will receive monetary compensation. Participants will be compensated for their time in the weigh-in and weigh-out sessions and performance-based incentives will be provided in the form of points which can redeemed for gift cards or toys at the end of the experiment. The experiment incurs no costs to the participants and both the children and parents will be compensated for their involvement along with any travel expenses incurred during the component of the experiment conducted at BCH.

1. Specific Aims /Objectives

| Specific Aim 1: The overarching goal is to lay the empirical and methodological foundation for developing a range of inhibitory control gaming interventions that offer alternatives or supplements to drug interventions for ADHD patients.  Specific Aim 2: To evaluate the suitability of neuroplasticity-based training modules for the special population of ADHD patients. One of the challenges facing computer based interventions for children with ADHD is the ability to drive user engagement given attention-related deficits in these patients. The first aim of this proposal is to run a behavioral pilot to identify design parameters most suitable for ADHD children.  Specific Aim 3: To evaluate the impact of focused inhibitory control training on ADHD behavioral symptoms as well laboratory measures of ADHD dysfunction. This experiment will use a strategy that integrates laboratory-based tests of executive function (EF) & electroencepholgram (EEG) physiology data with real-world (home and school-based) ADHD behavioral assessments to provide a multi-dimensional evaluation of the transfer of training effects. |
| --- |

2. Background and Significance

| Attention-deficit/hyperactivity disorder (ADHD) is a neurobehavioral condition marked by pronounced behavioral impairments including impulsivity, hyperactivity, and/or inattention. While its public health impact is disproportionately severe in the youth population, it is becoming increasingly documented that many patients (50-60%) suffer from its symptoms into adulthood, even in individuals for whom the diagnosis does not persist, the effect of a childhood diagnosis of ADHD on important life outcomes is severe (Barbaresi, Katusic, Colligan, Weaver, & Jacobsen, 2007; Yoshimasu et al., 2010). The first-line treatment of choice for most patients is pharmacological therapies (most notably amphetamine, methylphenidate and other psychostimulants) and clinical studies have established their efficacy in treating the acute behavioral symptoms of the disorder in the short and long term (Barbaresi, Katusic, Colligan, Weaver, & Jacobsen, 2007b; Molina et al., 2009; “National Institute of Mental Health Multimodal Treatment Study of ADHD follow-up,” 2004). While stimulant medications are effective interventions, patient compliance to treatment is often low over the long term (Barbaresi et al., 2006), and no intervention behavioral or pharmacological is effective in producing long-term changes ADHD symptoms when they have been discontinued. Thus, all current treatments effect change in symptoms while they are administered, but no treatment changes the deficit that theoretically gives rise to ADHD behaviors. In addition to this gap, stimulant medication in particular has idiosyncratic effects across patients, may have ‘rebound’ effects, and in adulthood may be associated with negative cardiovascular side effects. Exploration of new intervention avenues may decrease the reliance of young ADHD patients on continuing to take medication into adulthood while still targeting the behavioral deficits of the disorder.  Over the past two decades, neuroimaging and other systems-level neuroscience research has rendered a more complete picture of the specific neural systems (both structures and networks) that are compromised in ADHD (Durston, 2003). In particular, considerable attention has been paid to the possibility that ADHD symptoms result from a central deficit in inhibitory control. Isolating cortical and subcortical abnormalities associated with this cognitive deficit opens new doors for intervention. This research program evaluates neuroplasticity-based intervention technologies that foster plasticity in the neural circuits compromised in ADHD, specifically in inhibitory control. This research program outlines the plans to test the feasibility of these training interventions focusing on the fronto-striatal systems that underlie inhibitory control. Convergent evidence highlights this system as one of the focal sites of neural dysfunction in ADHD patients (Campbell & von Stauffenberg, 2009; Cubillo, Halari, Giampietro, Taylor, & Rubia, 2011).  Previous studies of computer training have been used to reduce symptoms in children with ADHD (Klingberg, Forssberg, & Westerberg, 2002) and increase learning in school (Holmes, Gathercole, & Dunning, 2009, 2010). In adults, training working memory is associated with improved performance on tests of fluid intelligence and changes in the function and structure of the prefrontal cortex(Jaeggi, Buschkuehl, Jonides, & Perrig, 2008; Klingberg, 2010; McNab et al., 2009). While training such as these have met with moderate success, no evidence currently exists from a study of a cognitive training study which directly addresses the central deficit in ADHD, inhibitory control.  Over the past three years, NeuroScouting LLC has developed and empirically validated diagnostic and training technologies that optimize control networks, specifically inhibitory control systems in the specialized population of professional baseball players – a group requiring deployment of these neural systems in game settings requiring the control of action. Research in pro sports has validated the efficacy of these methods to drive behavioral improvements on the field. This research program will build upon these exiting tools to optimize the efficacy and suitability of this technology for ADHD patients. |
| --- |

**3. Preliminary Studies**

| **Inhibitory Control Training in Elite Performers** Over the past five years, NeuroScouting has conducted leading research to develop and validate neural analysis and neuroplasticity-based training in professional athletics. During this time, over 7000 analysis and training sessions have been conducted with over 500 professional baseball players and the training technology is employed daily as a system-wide evaluation and training tool. NeuroScouting’s brain analysis tools sensitively measured small differences between elite professional baseball players and these diagnostics were strongly linked to inter-individual differences in key metrics of on-field plate discipline – a real-world behavior calling upon IC systems in the brain (left panel; decreasing variance was linked with higher isolated plate discipline – IsoD). While this skill requires a number of biomechanical, psychological, and neural processes, the strength of this link indicates that the brain networks underlying IC are critical contributors to the real-world behavioral ability displayed on the field. Also, neuroplasticity training during the 2010 and 2011 baseball seasons (with players training 3-4 times per week) showed that the amount of improvement in IC variance exhibited in NE training strongly correlated with on-field improvements (increased IsoD) from year to year (Right panel; note – unlike left panel, improved variance (a decrease) is plotted on the positive y-axis as a positive change in precision). Importantly (as shown in Figure 2), players that engaged regularly with the training system (black bar) showed a markedly larger increase in IsoD than players that trained less with the system (gray bar).  While elite athletes are not the same as children with ADHD, this research demonstrates several important and valuable points. First this is an example of excellent ‘far transfer.’ Practice at one thing (playing an inhibitory control computer task) is likely to improve performance on that task and tasks that are very similar to it (e.g., playing another kind of computer task). What is necessary to treat ADHD is, however, true strengthening of a core cognitive ability that can be deployed in novel situations. Neuroscouting has created just such a tool, as they demonstrate here by showing that practice with these computer games enhances on field performance, during games. Second, this evidence supports the central idea that inhibitory can be trained, and is in fact, trained in these computer games. |
| --- |
|  |

**4. Design and Methods**

**a. Study Design**

| Study Procedures for Experiment 1: In this short 60 minute behavioral session, three different experimental designs are tested to determine the parameters most suitable for the training modules used in Expt 2. All behavioral experiments will be performed on a laptop computer in the behavioral testing room at BCH. The primary goal of Experiment 1 is to determine optimal task parameters that balance the maximum number of trial events (to increase training exposure) with a task length that is manageable for ADHD patients. This experiment will focus on two design parameters: 1.) Pacing of trial events (i.e. inter-trial interval, ITI). 2.) Block length (length of contiguous trials before a break). Three sessions with different ITI/block length parameters will be delivered and both objective and subjective measures of user compliance and engagement will be recorded. The same behavioral task will be used for all trial conditions (see data collection methods below). While participants are playing this game, we will be recording EEG, this data will serve as pilot data that may help us know which neural markers are most informative about ADHD symptomology in this population. This information will be used to guide analysis of Experiment 2.  Study Procedures for Experiment 2, Stage 1: The first session of Expt 2 will involve a 2 hour evaluation session at the Laboratory of Cognitive Neuroscience at Children’s Hospital, Boston. A 6-minute recording of resting EEG will be followed by 3 short cognitive test modules measuring discrete executive functions (with EEG recorded throughout the session). This battery of tests will include 1.) an inhibitory control task, 2.) A switching task, and . 3.) Working Memory (for details of these tasks, see data collection methods). Baseline diagnostics will also include key metrics including the Diagnostic and Statistical Interview for Children (DISC-IV), the SNAP, and the Conner’s Symptom questionnaire, and the child behavior checklist (CBCL). Additionally at this time period, a Teacher Conner’s Form will be collected online. These diagnostics are aimed at evaluating behavioral deficits both in home and at school.  Following this training session participants will be randomized into either training (N=25) or control (N=25) game play. In the training game group, participants will be given a game that adaptively increases in difficulty, matching their learning. In the control group, participants will be given a game that does not change in difficulty as children play it. This control has been used in several studies of cognitive training, the adaptive nature of the training game is one of the central ‘ingredients’ for creating a successful intervention (Klingberg, 2010)  Study Procedures for Experiment 2, Stage 2: In the 4 weeks following the Diagnostic Session, Training Game participants will perform one 15 min long training module each day designed to train inhibitory control networks. These modules will be administered on tablet computers (iPads) that the patients will be provided for the duration of the training experiment. Structure of Training Modules: Participants will train 5 days / week with a different training game each week (participants choose their favorite training game for the final week). These training games will have design parameters (e.g. inter trial interval, block length) determined by the results of Expt 1 but will all have differences in gameplay to introduce novelty to increase user engagement/compliance (see data collection methods). During each week, the parameters constraining the range of latencies for each participant’s stop-signals will be set based on their performance gains and the slope from the previous day’s training session. This method ensures that each day the game meets the participant at their previous performance level.  In contrast, the control group will perform the same set of games except that these control games will be not be continuously adapted to the performance threshold of each individual. Instead, the games will be presented at a fixed set of demand levels ranging in equal difficulty increments from easy to hard and will involve no customization or adaptation to the individual.  Study Procedures for Experiment 2, Stage 3: The same diagnostics/EEG protocol employed in Stage 1 will also be conducted in the 1.5 hr follow-up diagnostic session with the exception of the CBCL or DISC. |
| --- |

**b. Patient Selection and Inclusion/Exclusion Criteria**

| **Inclusion Criteria**: All of the participants in these experiments will be American English speaking children between the ages of 8 and 11 years of age. The sample is limited to English speakers because experimenters will have to communicate with participant’s families extensively during the training, and for the pilot, the lab can only do this with English speakers. Starting the research program with a circumscribed age range will help to minimize age-related confounds in the behavioral performance and decrease the change of this variability becoming a pitfall for the experiments outlined. Another reason this age group was selected was to ensure participants have a relatively equal amount of video game experience. By keeping the age-range small, the experimental plan ensures less variable videogame histories that could be a confounding factor if a large age range with varied videogame histories were included in the experiment.  **Exclusion Criteria**: History of brain damage or other neurological complications, diagnosis of any disorder on the autism spectrum (ASD, PDD, Asperger’s Syndrome). Given the duration of the training interval that will be used in Expt 2 (and the desire for med status to be the same across Experiment 1 and 2) participants will be permitted to be on meds in Experiment 1 and during the training phase of Experiment 2. While med status, dosage, etc. will not be exclusion criteria, they will be recorded to track any systematic contribution of medication to the results. For the baseline and outcome measures in Experiment 2, we will request that they stop medication for 24 hours before their next study visit. Participants |
| --- |

**c. Recruitment Methods**

i. HOW, WHERE and WHEN will potential subjects be recruited?

| Children will be recruited from the Boston area through several key avenues: 1.) BCH’s IRB-approved Participant Recruitment Database (Protocol number 06-06-0261) set up by the Division of Developmental Medicine Laboratories of Cognitive Neuroscience. 2.) Recruitment fliers (example attached). 3.) Word-of-mouth, 4) identification of potential participants in the I2B2 database, and inviting those participants to be a part of the study.  1) The Participant Recruitment Database will be used at BCH, Boston to phone screen potential participants. As is done when using this database we will first send an 'opt-out' letter to parents inviting them to let us know if they are not interested in being contacted about this research opportunity. Next we will call parents who have not responded to the optout & invite them to participate in this study. If they are interested we will ask them to complete a short (5-10 min) phone screen on the phone.  2) We will post recruitment flyers. If parents respond to these flyers we will complete the phone screen.  3) Word of mouth. Sometimes participants hear of our study because they look at the BCH website or follow the LCN facebook page, or know anohter participant. If parents contact us because of one of these outlets we will complete the phone screen described above.  4) I2B2 List. We will identify children ages 8-11 years, with a current diagnosis of ADHD who are seen at Boston Children's Hospital and who have no history of any of our exclusion criteria. As is done when using the participant database we will first contact the physician of potential participants to ensure there is no reason a child should be excluded from the study. Then we will send an 'opt-out' letter to parents inviting them to let us know if they are not interested in being contacted about this research opportunity. Next we will call parents who have not responded to the optout & invite them to participate in this study.  All recruitment will be done by the PI or a research assistant the PI has trained directly.  The phone screen will consist of (A) a short contraindication screen; (B) the Swanson, Nolan, and Pelham (SNAP) ADHD screening questionnaire; (C) questions addressing willingness to participate in the one laboratory visit for Experiment 1 or two laboratory visits and 4 weeks of training in Experiment 2 and (D) willingness to name one other adult, not a family member, who knows the child well and may be willing to answer questions about the child, usually a teacher. Eligibility for full participation in the study will be a verified ADHD diagnosis. ADHD diagnosis will be confirmed by the SNAP and by contacting the child’s teacher (or other adult) before the first visit in both experiments and administering a diagnostic interview (Diagnostic Interview Schedule for Children; DISC-IV) to parents during their first visit in Expt 2. The gender distribution of this group of children is expected to reflect the distribution of gender in the ADHD population in general, approximately 2:1. Minorities will be recruited based on demographics of the greater Boston area. |
| --- |

ii. WHAT recruitment methods and materials (e.g. posters, fliers) will be used? *- attach all materials*

| Use of the subject databases, Flyers, word of mouth. Flyers, phone screens, physician contact emails/letters, and parent opt-out emails/letters are all attached. |
| --- |

iii. WHO will be responsible for subject recruitment?

| The principal investigator and associated study staff (research assistant) will be responsible for the subject recruitment. |
| --- |

**d. Description of Study Treatments or Exposures/Predictors**

| During this study participants will play one of two kinds of computer games which will constitute the treatment. Computer game 1 (treatment) will increase in difficulty as participants play the game. Computer game 2 (placebo) will remain at a constant level of difficulty. |
| --- |

**e.** Definition of Primary and Secondary Outcomes/Endpoints

| This study assesses training-related improvements in inhibitory control measures in tandem with electrophysiological changes pre and post training. 20 ADHD children will participate in Expt 1 which has the goal of determining task parameters that lead increased indices of sustained attention (in particular, the sharpest slope in the psychometric response curve). In Expt 2, 50 ADHD children will make 2 visits to BCH separated by a 4-week training protocol that they will complete at their homes. The BCH visits will administer baseline and outcome behavioral diagnostics while EEG is recorded. We expect 70 children total to participate, all with ADHD diagnosis in the age ranges of 8-11 years. |
| --- |

**f. Data Collection Methods, Assessments and Schedule** (what assessments performed, how often)

| Prior to scheduling the testing session (EEG and/or fMRI and Behavioral), the researcher will obtain consent prior for pre-screening. All families recruited through the LCN database or I2B2 database will be contacted after not receiving the “opt out” post card. At the first contact, we will obtain verbal consent before administering the pre-screeing questions and will receive formal written consent once they arrive at BCH Boston.  Researchers will schedule the sessions at a convenient time for the child and his/her parents. Parents and their children will participate at BCH, Boston and the researchers will explain the procedure and methods in detail. If the parent is willing to let his/her child participate, he/she will be asked to complete a consent form and several questionniares (above). Information volunteered by parents will only be identified by the participant numbers. All questionnaire information will be stored independently of consent forms in a locked file cabinet.  **Basic Behavioral Paradigm (Expt 1)** Expt 1 will utilize the training module from Expt 2. The gameplay is in the form of a simulated baseball environment but constitutes a classic go/no go or stop task paradigm. A moving ball is delivered on the screen (i.e. the ‘pitch’) and participants intercept it with a finger press on the tablet screen when it reaches a specific point on the screen (i.e. the simulated ‘home plate’). The target is pressed on every pitch (GO trials) unless the simulated ball changes color (red) during the course of its travel (STOP trials). Feedback points are accrued by either accurately intercepting the white balls (“a hit”) or appropriately inhibiting on the red balls (“a stop”). This feedback is displayed post-trial in the form of gold tokens (with different point totals) and can be used to receive gift cards at the end of the session. While the in-session reward will track with progress to continually motivate the participants, end-of-week bonus points for successful completion will equate the overall reward across all participants, thus no participant will be rewarded more than anyother. This game is a modified stop-signal (SS) task optimized to directly challenge inhibitory control abilities. inhibitory control tasks often conflate a range of executive function processes with inhibitory control demands. In order to minimize response-related demands (e.g. selection, motor control), the motor output in this module is identical on every trial and is kept extremely simple (e.g. a screen tap on the iPad). This also minimizes individual variability in response-related processes providing a higher fidelity measure of changes in inhibitory control function. Importantly, while this module uses a simple interface and finger press, theoreticaly, the same inhibitory control systems engaged control other effector systems (including manual, speech, and oculomotor systems) that accompany ADHD symptoms in the real world (Campbell & von Stauffenberg, 2009; Cubillo, Halari, Giampietro, Taylor, & Rubia, 2011). As a result, while this particular module involves sports-specific ‘gameplay’, it directly targets the neural systems involved in the inhibition of a range of actions in the real world. The algorithm controlling the module adapts throughout the session to adjust difficulty based upon performance. The difficulty increments are made in the form of slight adjustments in the input to the inhibitory control system (i.e., the latency of onset of the stop signal). *The proprietary algorithm driving the game was developed by NeuroScouting and works throughout the session to establish a stable estimate of each individual’s performance threshold.* This estimate is derived from a proprietary estimation method combining standard psychophysical methods with new advances in statistical analysis to reliably characterize each individual’s psychometric function. 3 short pilots of this will be administered to participants (5 min each): 1.) Short ITI (0.5s ITI)/Short Blocks (30s). 2.) Short ITI (0.5s ITI)/Long Blocks (2.5 min). 3.) Long ITI (3s ITI)/Long Blocks (2.5 min). Following the 3 session experiment, participants will fill out a survey asking for (Likert-measured) self-report on questions of engagement, subjective feeling of concentration, and preference amongst the three sessions.  **Executive Function Benchmarks (Expt 2, Stages 1 & 3)** The structure of the inhibitory control module will be discussed in detail as the key example, but all these modules were developed to convert neuropsychological batteries of executive function into more sensitive measures of individual difference points by including the same proprietary algorithms/estimation methods used to sensitively measure differences between previous participants. Inhibitory control diagnostic task: A benchmark inhibitory control will be employed to measure stop signal reaction time pre/post. This game is adapted from stop signal tasks employed in the literature and uses a visual stop signal within the context of a left/right arrow discrimination. Participants press the appropriate direction button based on the stimulus unless it flips upward at any point during the trial (in which case, they hold off). Administered at baseline and post-training, the task can track progress throughout training without being susceptible to practice effects that will confound progress within the training modules themselves. The latency of the stop signal will occur at a variable stop-signal delay after stimulus presentation controlled by the adaptive algorithm employed in Expt 1 to derive robust estimates of the psychometric function.  **Structure of Training Modules (Expt 2, Stage 2)** Details of the training game (TG) modules: TG1 – “Baseball Game”: Participants perform the same task laid out in Expt 1 – a simulated baseball environment with changes in the color of the ball indicating whether the participant should go or not go. TG2 - “Fill Game”: Participants will be viewing a glass filling with milk. They are instructed to indicate (i.e. touch the screen) when the milk reaches the top of the glass (but before it spills over). If the milk stops filling at any point, the participant is instructed to hold off on their response. TG3 – “Race car Game”: Participants are positioned in a racecar on a track. As they rapidly proceed through the track, participants are instructed to indicate with a touch when they identify any object (e.g. an oil slick) on the track. If those obstacles disappear at any point during the approach forward, they hold off. The final design of these training games may be informed by data collected in Expt 1, but any eventual task structure will be in the form of a simple videogame to engage these young patients.  **Standardized Measures.** In Expt 2, Stage 1, participants’ ADHD symptomology will be characterized with the Diagnostic and Statistical Interview for Children (DISC-IV), administered to each child’s parent or guardian while they are participating in the study. The DISC-IV is a diagnostic interview designed to assess psychiatric symptomology in children, only the ADHD sub-section will be administered. For all children, a primary caretaker will complete the Conners questionnaire, the SNAP, the BRIEF, and the CBCL, questionnaires about ADHD symptomology and daily executive function. In addition, an adult, who observes the child outside of the home setting, will complete a Teacher Conners Form designed to assess ADHD symptomology. During the Stage 3 final visit their parent or guardian will complete a Conners, SNAP, and BRIEF. The children’s teachers will also complete a follow-up Conners.  **Imaging methods and procedures at BCH: EEG.**Resting EEG data will be collected from participants at each visit. To do this, we will have children facing a computer monitor, they will be told to open their eyes and close their eyes at regular intervals for 6 minutes, allowing us to observe resting EEG in both eyes open and eyes closed conditions. In addition, EEG will be measured during the executive function diagnostics in the behavioral testing of Expt 2, stages 1 and 3.  EEG recording will be accomplished using a 128-channel Sensor Net System. The net is comprised of an elastic tension structure forming a geodesic tessellation of the head surface containing carbon fiber electrodes embedded in pedestal sponges.  At each vertex, there is a sensor pedestal housing an Ag/AgCl- coated, carbon-filled plastic electrode and sponge containing saline electrolyte. Prior to fitting the Sensor Net over the scalp, the sponges are soaked in electrolyte solution (6cc KCL/liter distilled water) in order to facilitate electrical contact between the scalp and the relevant electrode. Prior to recording, measurements of channel gains and zeros are taken to provide an accurate scaling factor for display of waveform data and so that baseline correction can be performed.  The child’s head is measured and marked in order to ensure accurate placement, and the net is then placed over the scalp.  Scalp impedances are checked on-line using NetStation, the recording software package that runs this system (EGI, Inc.). The advantage of this system is that it takes very little time (~10-15 minutes) to set up and children generally tolerate the net very well.  EEG data are collected and recorded on-line using NetAmps Amplifiers (EGI, Inc.) and the NetStation software. The data are amplified, filtered (band pass 0.1-100.00 Hz), and sampled at a frequency of 250 Hz.  They are digitized with a National Instruments Board (12 bit) and a Macintosh Power PC.  **Compensation and Travel**  As described in the consent forms, participants and their families will be compensated for their time if they come to Children’s Hospital Boston. Children will receive small toys or prizes, while families will be compensated $30 for Expt 1 and $140 for Expt 2. Families driving to BCH will have validated parking. To make it easier on families to travel to BCH, they will either be compensated for their travel at a set rate ($10) or have a car service pick them up from their homes and take them to Longwood. This will be arranged prior to the first visit and will be confirmed with the family during the reminder call the day before each visit. |
| --- |

g. Study Timeline (as applicable)

| Data will be collected over a 12-month period. |
| --- |

**h.** Adverse Event Criteria and Reporting Procedures

| No adverse events are expected for this study. In the case of an injury or extreme negative reaction to the testing procedures on the part of either the participant or his/her parent, an adverse event report will be filed with the IRB immediately. |
| --- |

**i. If the Investigator is the Sponsor/Assignee (IND or IDE-holder), he/she is responsible for selecting a qualified monitor who will monitor the progress of all clinical investigations conducted under the IND or IDE. Please describe the monitoring plan for this protocol below:**

⮡ Note: the EQuIP office provides monitoring services and advice. For info, contact EQuIP @ 5-7052.

x Not applicable

**5. Data Management and Statistical Analysis**

**a.** Data Management Methods

| The data will be collected by the PI, post-doctoral fellows, and research assistants at the Developmental Medicine Center Laboratory of Cognitive Neuroscience. Behavioral and EEG data will be stored on secure server space at BCH. Access will be provided only to staff directly involved with the project. All information linking the participants’ names to their unique subject identifier will be stored on a secure server at Boston Children’s Hospital. Any hard copies will be stored in locked file cabinets in a secure storage area. De-identified behavioral data will be stored on Neuroscouting’s secure servers at their headquarters in Cambridge, MA. |
| --- |

b. Quality Control Method

| The staff collecting data will be extensively trained in the use of Neuroscouting’s behavioral tests and imaging equipment. Quality control will be ensured by regularly maintaining equipment and data will be processed as it is collected to allow the early identification of potential problems with data quality. There will be no personal identifying information attached to the data after it is collected. Researchers and research staff will be blinded to participant category (control vs. training) when analyzing and interpreting the data that may be subject to a bias. We will also ensure quality control also be ensured by assessing reliability between examiners on the behavioral tests and EEG. |
| --- |

**c. Data Analysis Plan**

| **Behavior Links with ADHD Symptomology:** The core analyses will examine if there is a link between training-related behavioral changes (as measured by executive function pre/post measures) and ADHD symptoms. To determine if there is change in ADHD symptoms and improvement in task performance across time we will first (Model 1) look for group differences (control vs. intervention) in all EF pre/post measures but will focus specifically on inhibitory control performance using multiple regression, controlling for age, gender and inhibitory control performance at baseline to determine if participants improve in task performance. Next, (Model 2) we will perform the same analysis using ADHD symptoms (average severity from SNAP & Conners) as a dependent measure: we will observe group (control vs. intervention) effects on ADHD symptomology controlling for age, gender, and ADHD symptoms at baseline. Finally, (Model 3) to determine if changing inhibitory control parameters mediates the effect of our intervention on ADHD symptoms, we will run Model 2 additionally controlling for the residual from Model 1 from the group effect on inhibitory control perofrmance. We expect that the group effect on inhibitory control parameters specifically will mediate the effect of group on ADHD symptomology that we will operationalize as a significant decrease in the association between group and ADHD symptoms between Model 2 and Model 3. We will measure the significance of this mediation model using a non-parametric bootstrapping approach to estimate the distribution of the change in effect of each predictor on the outcome after controlling for the mediators (3000 bootstrap resamples) as described in Preacher & Hayes, 2004 (see also, Shrout & Bolger, 2002). Generating this distribution allows the construction of a confidence interval for a particular model from which significance can be determined. This approach has been suggested for use with smaller sample sizes because it does not rely on assumptions of normality (as does Sobel’s test), which are often violated in smaller samples. In addition, this approach can account for decreases in significance that result from including an additional predictor in a regression model with a small sample size (Hayes, 2009).  **Data Analysis: EEG**. First, all EEG channels will be filtered and then re-referenced to the average reference. We will extract at least 120 continuous seconds of EEG recording, and then export these data to Matlab for further analysis.  Using EEGlab (open source Matlab toolbox), we will visualize the data and remove periods of movement artifact.  We will then use the first 90 seconds of artifact free data for further analysis.  Second, we will calculate the power spectral density in Matlab using a Hanning window with a length of 0.25Hz, overlapping by 50%. To plot and analyze the data, we will take the log_10_ of the power spectra to normalize the data.  Consistent with prior research specifying EEG frequency bands in this age range, spectral power in the following frequency bands will be computed: theta (3–5 Hz), alpha (6–9 Hz), and beta (10–18 Hz). Relative power (RP) in each frequency band is computed as the proportion of power at a given electrode site relative to total power (3–18 Hz) at that same electrode site. Relative power minimizes individual differences in EEG absolute power resulting from variations in age at assessment as well as skull thickness and other anatomical factors. Analysis will focus on the left- and right-sided electrodes over the frontal (electrodes on and near 10-20 system F3, F4), central (C3, C4), parietal (P3, P4), occipital (O1, O2), and temporal (T7, T8). In addition to traditional analysis, novel EEG analyses will be employed. ERP peak latencies and amplitudes will be obtained from appropriate scalp sites over pre-selected latency ranges. In addition to these analyses, large-scale dynamics will be measured using a recent variant of time-frequency coherence, *Phase Coupling Estimation* – a multivariate analysis technique which measures phase coupling across electrodes to quantify the spatial and temporal nature of network interactions (56,57). Additionally, novel machine learning analyses will be applied to the EEG data to identify unique dependent measures that can be used to sensitively investigate training-related changes. These exploratory analyses will be conducted to explore training-related changes in ERP, spectral, and functional connectivity patterns to investigate the neural basis of pre/post behavioral differences and to highlight dependent measures to utilize in future Phase II research work. |
| --- |

d. Statistical Power and Sample Considerations

| In choosing the number of the participants for the investigation, the subject numbers were selected to balance statistical power with a subject number that can be realistically collected within the 12-month time window of the research. We also conducted a power analysis that included an estimate of moderate effect size (f=0.25) based upon the training effects observed with this technology in over 7000 samples in non-ADHD athletic and control populations. In this analysis, inclusion of 25 patients in each experimental condition met the criterion power of 0.80. This sample size also mirrors other investigations that successfully explored working memory training in ADHD in a randomly controlled manner (e.g. N=53 split between training and control in Klingberg et al., 2005). |
| --- |

e. Study Organization

| The study PI, Margaret Sheridan, will be responsible for overseeing data collection, processing, and analysis. Research assistants at BCH, Katrina Bridgman, Kelly Khem, and Warren Winter, will be in charge of patient recruitment, data collection, and interfacing with patients and their families to ensure compliance during the training period. Staff at Neuroscouting will provide the relevant software and training on how to administer these tasks to participants. |
| --- |

**6. Risks and Discomforts**

| The methods employed in this proposal are based on behavioral methods that have been widely employed in previous cognitive and brain research in children (Holmes, Gathercole, & Dunning, 2009, 2010). The risks of participating in the experiment are no greater than the risks incurred with working at a computer for 30 minutes and therefore are no greater than minimal risk to the children. The training phase of experiment will be conducted at a time of the participant’s choosing at a location of their choosing to maximize comfort. Breaks will be included to prevent participant fatigue. At the end of the session, the participant will have the option to submit their data or to not submit their data. This ensures that they are able to withdraw from the experiment at any time if it is causing discomfort, fatigue, etc.  For the EEG sessions proposed in Expts 1 & 2, there are minimal foreseeable risks and discomforts to the participants arising from participation in this research study. The time commitment for this study is as follows: 20 15-minute training sessions (no EEG) and two 60-minute pre/post measurement (with EEG) and interview sessions. Assessments will be conducted at LCN at Children’s Hospital, Boston and training will be conducted at the participants’ homes.  Loss of confidentiality is always a risk in studies using human subjects. In this study that risk is minimal because all diagnostic phases of the experiments will be conducted in a private testing room in Children’s Hospital Boston. The primary research material collected in these two experiments will be in the form of scores reflecting the performance and EEG signatures of each individual in the analysis and training behavioral tasks. Additionally, behavioral assessments of ADHD will be recorded for each participant.  To ensure confidentiality, the following steps will be taken:  1. All consent forms, once signed by parent and child will be placed into a manila envelope and kept with the experimenter. The forms will be placed in the same locked file cabinet at BCH after the experimental testing session is complete.  2. All of these data materials will be collected on a secure online connection (training phase of Expt 2) or on secure systems in Children’s Hospital Boston (Expt 1 and baseline / outcome assessments of Expt 2) and stored on a dedicated server. The only individuals with access to the private information about the participants will be the principal investigators and key personnel.  3. For collection of data from teacher’s in the Conners, a secure portal will be established on BCH’s server. The teacher will be supplied a unique url (which contains no information about the participant’s name or any link to their subject ID) to complete the form. Upon submission, the url will be destroyed and data housed securely on the server.  4. All subject’s data will be identified only with a subject number. Once the child has participated, their data will not be linked with their identity.  5. Children and parents will be allowed to stop at any time.  **REFERENCES**  Barbaresi, W. J., Katusic, S. K., Colligan, R. C., Weaver, A. L., & Jacobsen, S. J. (2007a). Long-term school outcomes for children with attention-deficit/hyperactivity disorder: a population-based perspective. *Journal of developmental and behavioral pediatrics: JDBP*, *28*(4), 265–273. doi:10.1097/DBP.0b013e31811ff87d  Barbaresi, W. J., Katusic, S. K., Colligan, R. C., Weaver, A. L., & Jacobsen, S. J. (2007b). Modifiers of long-term school outcomes for children with attention-deficit/hyperactivity disorder: does treatment with stimulant medication make a difference? Results from a population-based study. *Journal of developmental and behavioral pediatrics: JDBP*, *28*(4), 274–287. doi:10.1097/DBP.0b013e3180cabc28  Barbaresi, W. J., Katusic, S. K., Colligan, R. C., Weaver, A. L., Leibson, C. L., & Jacobsen, S. J. (2006). Long-term stimulant medication treatment of attention-deficit/hyperactivity disorder: results from a population-based study. *Journal of developmental and behavioral pediatrics: JDBP*, *27*(1), 1–10.  Campbell, S. B., & von Stauffenberg, C. (2009). Delay and inhibition as early predictors of ADHD symptoms in third grade. *Journal of Abnormal Child Psychology*, *37*(1), 1–15. doi:10.1007/s10802-008-9270-4  Cubillo, A., Halari, R., Giampietro, V., Taylor, E., & Rubia, K. (2011). Fronto-striatal underactivation during interference inhibition and attention allocation in grown up children with attention deficit/hyperactivity disorder and persistent symptoms. *Psychiatry Research*, *193*(1), 17–27. doi:10.1016/j.pscychresns.2010.12.014  Durston, S. (2003). A review of the biological bases of ADHD: what have we learned from imaging studies? *Mental Retardation and Developmental Disabilities Research Reviews*, *9*(3), 184–195. doi:10.1002/mrdd.10079  Holmes, J., Gathercole, S. E., & Dunning, D. L. (2009). Adaptive training leads to sustained enhancement of poor working memory in children. *Developmental Science*, *12*(4), F9–15. doi:10.1111/j.1467-7687.2009.00848.x  Holmes, J., Gathercole, S. E., & Dunning, D. L. (2010). Poor working memory: impact and interventions. *Advances in Child Development and Behavior*, *39*, 1–43.  Jaeggi, S. M., Buschkuehl, M., Jonides, J., & Perrig, W. J. (2008). Improving fluid intelligence with training on working memory. *Proceedings of the National Academy of Sciences*, *105*(19), 6829–6833. doi:10.1073/pnas.0801268105  Kessler, R. C., Adler, L., Barkley, R., Biederman, J., Conners, C. K., Demler, O., Faraone, S. V., et al. (2006). The prevalence and correlates of adult ADHD in the United States: results from the National Comorbidity Survey Replication. *The American Journal of Psychiatry*, *163*(4), 716–723. doi:10.1176/appi.ajp.163.4.716  Klingberg, T. (2010). Training and plasticity of working memory. *Trends in Cognitive Sciences*, *14*(7), 317–324. doi:10.1016/j.tics.2010.05.002  Klingberg, T., Forssberg, H., & Westerberg, H. (2002). Training of working memory in children with ADHD. *Journal of Clinical and Experimental Neuropsychology*, *24*(6), 781–791.  McNab, F., Varrone, A., Farde, L., Jucaite, A., Bystritsky, P., Forssberg, H., & Klingberg, T. (2009). Changes in cortical dopamine D1 receptor binding associated with cognitive training. *Science (New York, N.Y.)*, *323*(5915), 800–802. doi:10.1126/science.1166102  Molina, B. S. G., Hinshaw, S. P., Swanson, J. M., Arnold, L. E., Vitiello, B., Jensen, P. S., Epstein, J. N., et al. (2009). The MTA at 8 years: prospective follow-up of children treated for combined-type ADHD in a multisite study. *Journal of the American Academy of Child and Adolescent Psychiatry*, *48*(5), 484–500. doi:10.1097/CHI.0b013e31819c23d0  National Institute of Mental Health Multimodal Treatment Study of ADHD follow-up: 24-month outcomes of treatment strategies for attention-deficit/hyperactivity disorder. (2004).*Pediatrics*, *113*(4), 754–761.  Yoshimasu, K., Barbaresi, W. J., Colligan, R. C., Killian, J. M., Voigt, R. G., Weaver, A. L., & Katusic, S. K. (2010). Gender, attention-deficit/hyperactivity disorder, and reading disability in a population-based birth cohort. *Pediatrics*, *126*(4), e788–795. doi:10.1542/peds.2010-1187 |
| --- |
