# Supplementary material for: Computer-based inhibitory control training in children with Attention-Deficit/Hyperactivity Disorder (ADHD): Evidence for behavioral and neural impact
Source: PLoS One. 2020 Nov 30;15(11):e0241352. doi: 10.1371/journal.pone.0241352 (PMC7703966; doi:10.1371/journal.pone.0241352)
Supplement: Attachment — Submitted filename: Response to Reviewers.docx [file pone.0241352.s007.docx]

**Response to Reviewers**: for manuscript **#PONE-D-19-18201,** “Computer-based inhibitory control training in children with attention-deficit/hyperactivity disorder (ADHD): Evidence for behavioral and neural impact” by Meyer, Santillana, Miller, Clapp, Way, Bridgmann-Goines, & Sheridan.

Dear Dr. Fernandez,

Please find attached a revised version of this manuscript incorporating the feedback and suggestions from Reviewer 3. There were no further comments from Reviewer 1 and Reviewer 2. Below are our detailed responses to the points raised. The reviewer’s comments are shown *in italics*, and our replies are directly below each comment with reference to the sections edited.

In addition to the specific comments by the reviewers, documented below, we read carefully the editors comments,

*“I consider several of the Reviewer3's suggestions to be important for improving the article. In particular: a) include in the Introduction the existing controversy regarding the absence versus presence of results when cognitive training is applied (maybe differences in treatment efficacy is related to training time, number of sessions or other variables that have not been made explicit in the document) and b) be very careful when making statements like "the first study of its kind to use computerized training of IC in children with ADHD"; Reviewer3 indicates that this is not the case, please carefully review the literature.”*

Thank you. We have included further discussion of the existing controversy in the introduction (p5 – p6), in which we address the heterogeneity of findings and incorporate findings from meta-analyses of cognitive training. We have also carefully reviewed the document to ensure we do not claim to be the first study to train IC in children with ADHD. Additionally, we have added discussion of the studies suggested by Reviewer 3 in our section of the introduction reviewing previous work of computerized cognitive training in ADHD (p5 and p6).

***Reviewer #3:***

1. *The authors might consider referencing the following related work and toning down the language related to the novelty of the present study and should consider referencing Shavlev, Tsal, & Mevorach (2002; they may have more recent work as well) and Jones, Katz, Buschkuehl, Jaeggi & Shah (2018). Specifically, in the abstract, the authors say that theirs is the only study in which cognitive training for ADHD has targeted inhibition. This is not the case.*

**REPLY:** We appreciate this reviewer providing further examples of work that has utilized cognitive training to target inhibition, among other processes, in children with ADHD, and we have reviewed this work in included the suggested citations on pages 5 and 6. Additionally, we carefully reviewed the manuscript and have removed any claims of being the first study to target IC in children with ADHD.

2. *In general, the authors do a good job describing their study and their analyses. However, most of the results are null. This is not a bad thing -- we should certainly publish such findings. However, the authors should be more upfront about this in the abstract, so as not to mislead the reader. Relatedly, the introduction section would do well to discuss that a large portion of cognitive training research has produced null or small effects. There are several metaanalyses showing both that cognitive training works and that it does not. I recommend that the authors acknowledge this ongoing debate and position their work as contributing to this ongoing discussion.*

**REPLY:** Thank you. We agree that transparency of null or small effects are important, and have included a report of null effects in the abstract. Additionally, we now include discussion of the ongoing debate regarding the effect sizes and transfer of cognitive training in our introduction (pages 5 and 6), including reviews of relevant meta-analyses.
